# Emerging DNA Methylome Targets in FLT3-ITD-Positive Acute Myeloid Leukemia: Combination Therapy with Clinically Approved FLT3 Inhibitors

**DOI:** 10.1007/s11864-024-01202-7

**Published:** 2024-05-02

**Authors:** Melisa Tecik, Aysun Adan

**Affiliations:** 1 Bioengineering Program, Graduate School of Engineering and Science, Abdullah Gul University, Kayseri, Turkey; 2 Department of Molecular Biology and Genetics, Faculty of Life and Natural Sciences, Abdullah Gul University, Kayseri, Turkey

**Keywords:** FLT3-ITD AML, FLT3 inhibitor, Epigenetic therapy, DNA methylation, Combinational therapy

## Abstract

The internal tandem duplication (ITD) mutation of the FMS-like receptor tyrosine kinase 3 (*FLT3-ITD*) is the most common mutation observed in approximately 30% of acute myeloid leukemia (AML) patients. It represents poor prognosis due to continuous activation of downstream growth-promoting signaling pathways such as STAT5 and PI3K/AKT. Hence, FLT3 is considered an attractive druggable target; selective small FLT3 inhibitors (FLT3Is), such as midostaurin and quizartinib, have been clinically approved. However, patients possess generally poor remission rates and acquired resistance when FLT3I used alone. Various factors in patients could cause these adverse effects including altered epigenetic regulation, causing mainly abnormal gene expression patterns. Epigenetic modifications are required for hematopoietic stem cell (HSC) self-renewal and differentiation; however, critical driver mutations have been identified in genes controlling DNA methylation (such as *DNMT3A*, *TET2*, *IDH1/2*). These regulators cause leukemia pathogenesis and affect disease diagnosis and prognosis when they co-occur with *FLT3-ITD* mutation. Therefore, understanding the role of different epigenetic alterations in *FLT3-ITD* AML pathogenesis and how they modulate FLT3I’s activity is important to rationalize combinational treatment approaches including FLT3Is and modulators of methylation regulators or pathways. Data from ongoing pre-clinical and clinical studies will further precisely define the potential use of epigenetic therapy together with FLT3Is especially after characterized patients’ mutational status in terms of FLT3 and DNA methlome regulators.

## Introduction

Acute myeloid leukemia (AML) is a heterogeneous clonal disease characterized by aberrant proliferation of poorly differentiated cells in the hematopoietic system [1]. Patients are characterized based on the genetic alterations such as amplifications, deletions, rearrangements, and point mutations. The cytogenetic profiles of the patients are important in prognosis to assess risk levels and to decide on treatment approaches [2]. Some of the common gene mutations in AML are *FLT3*, *NPM1*, *DNMT3A*, and *N/KRAS*, considered significant diagnostic and/or prognostic markers [3, 4].

The FMS-like tyrosine kinase 3 (*FLT3*) encodes a receptor tyrosine kinase (RTK), which is mainly expressed on immature hematopoietic progenitors and hematopoietic stem cells (HSCs) [5•]. As these cells mature, the presence of the FLT3 receptor is reduced or completely lost [6]. FLT3 signaling is dependent on the binding of FLT3 ligand (FLT3L) to FLT3, resulting in dimerization of the receptor and autophosphorylation of the tyrosine residues. Then, downstream signaling pathways including PI3K/AKT, MAPK, and JAK2/STAT5 are activated, leading to increased cell growth and decreased apoptosis [7]. Two main mutations in the FLT3 receptor are internal tandem duplication (ITD) and tyrosine kinase domain (TKD) mutations, accounting for 20–25% and 5–10% of newly diagnosed AML patients, respectively [8]. These mutations cause FLT3L-independent dimerization and continuous activation of the receptor, initiating the aforementioned downstream signaling pathways, thereby increasing cell proliferation and suppressing apoptosis [1, 8]. *FLT3-ITD* mutations are associated with lower overall survival (OS) rate, poor treatment response, and shorter disease-free survival (DFS), which makes FLT3 an appealing treatment target in AML [9, 10]. Several small molecule inhibitors named FLT3 inhibitors (FLT3Is) have been developed with promising preclinical and clinical outcomes, some of which, including midostaurin, sorafenib, gilterinitib, and quizartinib, have been clinically approved for different clinical settings [11].

Pathogenesis of *FLT3-ITD* AML is not only triggered by this specific mutation but also by several epigenetic alterations [12]. Epigenetics alters gene expression without any change in the DNA sequence, which mainly includes DNA methylation, histone modification, and chromatin remodeling and impacts cell growth and disease development [13–15]. Dysregulation of epigenetic mechanisms activates oncogenes, inhibits tumor suppressor genes, and destabilizes the chromosomes, which leads to the development and progression of cancer [16]. Hence, several epi-mutations are commonly found in *FLT3-ITD* AML, including the gene mutations in histone modification Enhancer of Zeste Homologue 2 (*EZH2*) and the additional sex combs-like gene (*ASXL1*), regulation of DNA methylation (*DNMT3A, TET2*), and enzymes regulating metabolism (IDH1/2) with epigenetic consequences [14, 17]. Since epigenetic changes are reversible, targeting epigenetic regulators is thought to be a promising strategy in AML, such as using hypomethylating agents (HMAs), 5-azacitidine and decitabine, and histone deacetylase (HDAC) inhibitors [14]. However, the therapeutic efficacy of HMAs and HDAC inhibitors are limited when used as single agents. Hence, combination strategies of epigenetic therapy with targeted therapies such as FLT3Is are currently at different stages of pre-clinical and clinical studies.

In this review, the importance of DNA methylation in healthy and abnormal AML hematopoiesis will be summarized. Moreover, pre-clinical and clinical data regarding the role of DNA methylome alterations in the pathogenesis and treatment modalities of *FLT3-ITD* AML and potential approaches to target DNA methylation regulators alone or in combination with FLT3Is used in the clinic will be discussed.

## FLT3-ITD AML

### Molecular biology of FLT3-positive AML

The human *FLT3* gene is found on chromosome 13q12, containing 24 exons and 993 amino acid residues and encodes for FLT3 transmembrane receptor belonging to type III RTK family [8, 18, 19]. Major cell types expressing FLT3 receptor are HSCs, multipotent progenitors, most myeloid and lymphoid progenitor cells, and mature dendritic cells [20]. Each FLT3 receptor contains an extracellular domain made up of 5 immunoglobulin-like subdomains, a transmembrane domain, an intracellular juxtamembrane (JM) domain, and an intracellular C-terminal domain [8]. The intracellular domain is composed of tyrosine kinase domain 1 (TKD1) and tyrosine kinase domain 2 (TKD2), which are linked by an activation loop (A-loop) [8].

FLT3L is an extracellular ligand being either attached to the cell membrane or in a soluble state, which is mainly produced by lymphocytes, HSCs, and bone marrow stromal cells [21]. FLT3L concentration is generally low but can increase due to aplasia, ensuring the controlled activation of FLT3 via negative-feedback mechanism [22]. When FLT3L binds to the extracellular domain of the receptor, FLT3 receptor undergoes dimerization followed by conformational changes in the JM domain, making the kinase domain accessible for ATP binding, and consequent autophosphorylation and activation of the receptor [10, 18, 23].

Two major *FLT3* mutations, ITD and TKD, account for approximately 30% of AML patients, making them the most frequently identified mutations in AML [5•]. These mutations cause FLT3L-independent dimerization and activation of FLT3 receptor, hence, resulting in aberrant proliferation of the malignant cells even in the absence of FLT3L [24]. *FLT3-ITD* mutations occur in the JM domain, involving duplications of a fragment which vary in length and position [25, 26]. *FLT3-ITD* mutations are associated with increased relapse and reduced OS and the length of the duplicated fragment is inversely correlated with the OS [11, 25, 27]. On the other hand, *FLT3-TKD* mutations generally consist of single amino acid substitutions, deletions, or insertions located in the A-loop of the TKD, resulting in the loss of auto-inhibition [28]. Several signaling pathways regulating proliferation, differentiation, and apoptosis of HSCs are activated upon FLT3 dimerization [21]. Upon FLT3 binding, a series of events are triggered including autophosphorylation of tyrosine residues followed by adaptor proteins (such as GRB2, SHP2, and SRC family kinases) binding. These interactions primarily lead to the activation of PI3K/AKT/mTOR and RAS/MEK/ERK pathways [29–33]. *FLT3-ITD* and *-TKD* mutations activate similar pathways; however, *FLT3-ITD* specifically induces the JAK/STAT pathway through phosphorylation of STAT5A. Furthermore, FLT3-ITD mutations cause the reduced expression of C/EBPalpha and PU.1, which are crucial transcription factors for myeloid cell differentiation [34].

### Clinically approved FLT3 inhibitors

Several tyrosine kinase inhibitors (TKIs) to target mutant FLT3 have been investigated. However, only some of them have been approved for the treatment of *FLT3-ITD* AML including midostaurin, sorafenib, gilterinitib, and quizartinib [5•]. TKIs are categorized as first- and next-generation inhibitors, distinguished by their particular capacity to inhibit FLT3 and the related downstream cascades [10, 11]. These inhibitors can be further characterized as type I or type II inhibitors depending on their effectiveness against both *FLT3-ITD* and *-TKD* mutations, or solely against *FLT3-ITD* mutations, respectively [10, 11, 35].

*Midostaurin* is a first-generation, type I FLT3I, targeting both *FLT3-ITD* and *-TKD* mutations [5•]. Midostaurin is originally a protein kinase C inhibitor; however, it also inhibits other tyrosine kinases including FLT3 [36]. After recognition of *FLT3* mutations in AML pathogenesis, midostaurin is characterized as a FLT3i based on in vitro and in vivo studies [37]. Reports have shown that midostaurin inhibits FLT3 receptor signal transduction and induces cell cycle arrest and apoptosis [36]. In early clinical trials, midostaurin showed limited and transient activity as a single-agent treatment and induced about a 50% reduction in peripheral and bone marrow blast counts in relapsed/refractory (R/R) AML patients with *FLT3* mutation [38]. When patients carrying either wild-type FLT3 or mutated FLT3 were treated, midostaurin achieved 42% and 71% reduction in the peripheral and bone marrow blasts, respectively [39]. Therefore, midostaurin failed to induce complete remission (CR). The limited clinical efficacy of midostaurin is resulted from the activation of alternative pathways, protection of leukemic clones, and limited presence of free midostaurin in the plasma [38, 39]. However, combination of midostaurin with other cytotoxic agents showed promising results in in vitro models followed by clinical trials [40, 41]. Combination of midostaurin with standard chemotherapy in newly diagnosed younger patients with *FLT3*-mutated AML achieved high CR and high OS rates [42]. The milestone RATIFY (NCT00651261) trial led to approval of midostaurin by Food and Drug Administration (FDA) in 2017 with the results of significantly increased OS and reduced death by 22% regardless of the high or low mutant allelic fractions or presence of TKD mutation [43, 44].

*Sorafenib* is a first-generation, type I FLT3I originally developed as a RAF kinase inhibitor. Sorafenib has also shown activity against other tyrosine kinases including FLT3 [45]. In vitro studies reported that sorafenib inhibits phosphorylation of downstream target proteins of FLT3 including RAF, MEK, ERK, and STAT5A and induces apoptosis in a Bim-dependent manner [45–47]. SORMAIN study demonstrated that addition of sorafenib results in higher probability of 24-month OS [48••]. Sorafenib showed safe and efficient profile on FLT3 mutant AML when it is combined with standard anthracycline/cytarabine induction therapy [49]. In the study including 99 newly diagnosed *FLT3-ITD* AML patient, sorafenib plus intensive chemotherapy showed better OS [50]. The results of these clinical studies resulted in approval of sorafenib by National Comprehensive Cancer Network (NCCN) in 2019 [51].

*Gilteritinib* is a type I, next-generation FLT3I having more potency against mutated FLT3. The mechanism of action of gilteritinib is through binding of the drug to the active site of the receptor and stabilizing the inactive conformation, thereby inhibiting the downstream signaling molecules including ERK and STAT5 [52]. FDA approval of gilteritinib in 2018 for the treatment of R/R AML FLT3 patients was based on ADMIRAL trial (NCT02421939) which reported higher CR and OS rates compared to salvage chemotherapy. In this trial, 49 patients treated with gilteritinib lived more than 2 years [53, 53, 54].

*Quizartinib*, a next-generation, type II FLT3i showed high potency, precise kinase selectivity, and favorable pharmacokinetic properties in in vitro and in vivo studies, making it a great candidate for clinical trials [55, 56]. QUANTUM-R (NCT02039726), which is a phase III study assessing quizartinib as a monotherapy in R/R *FLT3-ITD* AML patients, resulted in increased OS compared to salvage chemotherapy [57••]. Thirty-two percent of patients treated with quizartinib arm could proceed to an allogeneic stem cell transplantation (allo-SCT), which was 11% in salvage chemotherapy group. However, quizartinib caused cardiac toxicities and strong myeloid suppression which prevented its approval by FDA in the USA although it is approved as monotherapy in R/R FLT3-ITD AML in Japan in 2019 [58]. In 2023, FDA approved quizartinib based on the results from the QuANTUM-First (NCT02668653), a phase 3 clinical trial, to be used as an induction therapy with standard chemotherapy, cytarabine consolidation therapy, and as maintenance monotherapy, despite its side effects [59].

### Epigenetics in FLT3-ITD AML

Cancer arises from a series of disruptions to the regulation of various cellular processes, such as cell growth, immortality, angiogenesis, cell death, invasion, and metastasis. Persistent changes in these functions are often driven by genetic mechanisms like mutations, copy number alterations, insertions, deletions, and recombination. As a result, cancer has traditionally been considered primarily a genetic disease. However, it is now clear that epigenetic changes offer an alternative route for acquiring stable oncogenic characteristics [12].

**Fig. 1:**
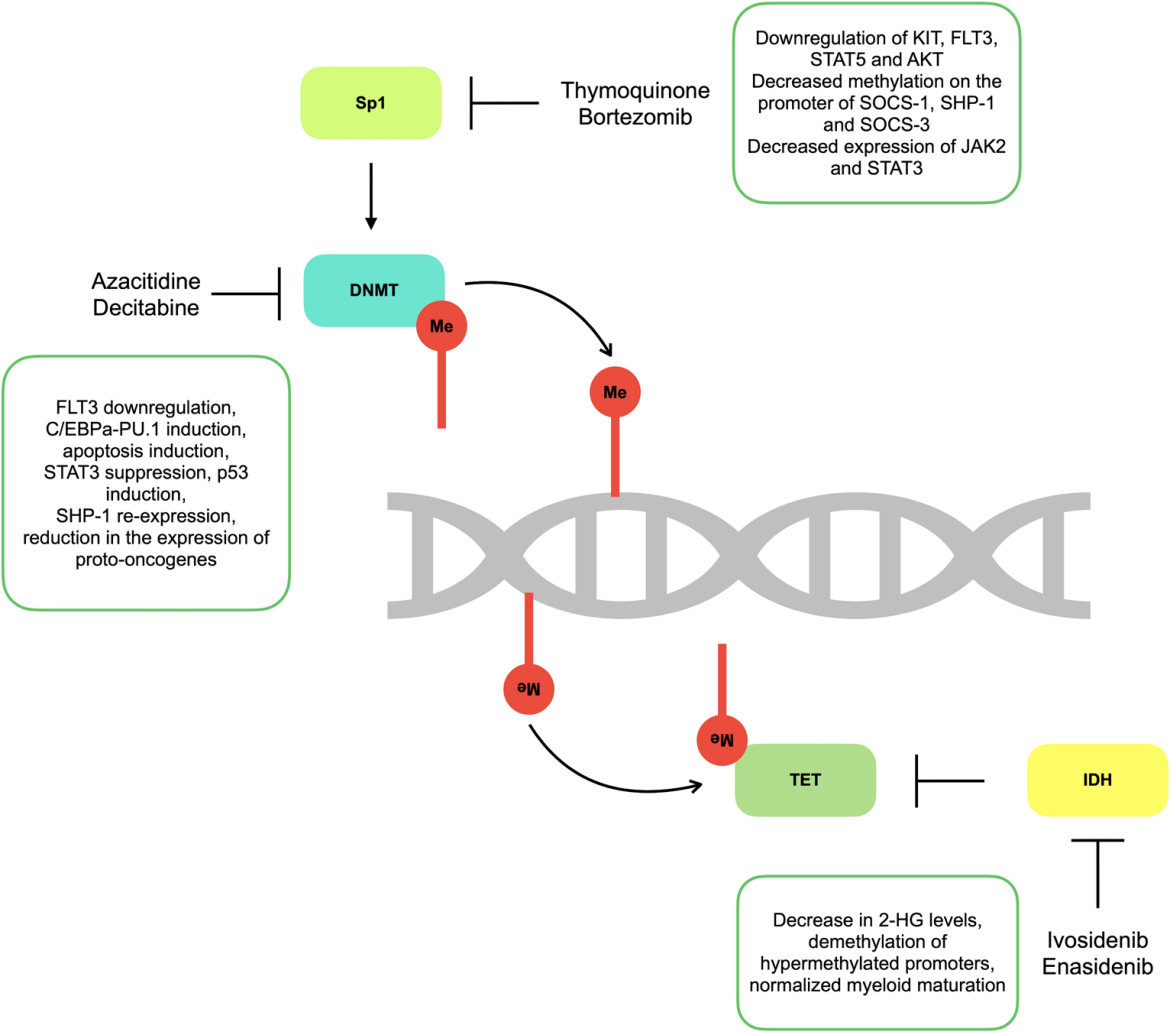
Mechanisms of action of DNA methylation targeting involved in FLT3-ITD AML pathogenesis. This figure summarizes new therapeutic strategies targeting DNA methylome in FLT3-ITD AML.

Epigenetics is commonly described as a genomic mechanism that exerts reversible effects on gene expression without changing DNA sequence [13]. These mechanisms involve DNA methylation, histone modification, chromatin remodeling, and RNA-associated modifications [14]. Epigenetic changes have the ability to influence cellular phenotype and to control several cellular activities such as cell growth, differentiation, and disease development [15]. Dysregulation of epigenetic mechanisms plays a crucial role in the development and progression of cancer, as they could activate oncogenes, cause chromosomal instability, and silence tumor suppressor genes [16]. Moreover, recurrent somatic alterations in genes with crucial roles in epigenetic regulation are frequently seen in AML [17]. Clinical observations and experimental evidences show that epigenetic mutations contribute to a pre-leukemic state but not adequate to cause full-blown acute leukemia [60]. Notably, the frequency of epigenetic mutations correlates with increasing age of AML patients. These mutations are also used as markers of prognostic risk stratification in AML [61]. As the changes caused by epigenetic mutations in the genome are frequently reversible, targeting epigenetic regulators or pathways holds important implications for therapeutic approaches in a specific subtype of AML, *FLT3-ITD* AML (Fig. 1) [14].

## DNA methylome modifiers and their roles in FLT3-ITD AML

### DNA methylation regulators in healthy and abnormal hematopoiesis

DNA methylation is a significant epigenetic modification in eukaryotic cells with a crucial role in regulating gene expression [62]. It is involved in several processes such as embryonic patterning, X-inactivation, and genomic imprinting [63]. The control of gene expression is achieved through addition of a methyl group to cytosine residues at specific CpG islands found in the promoter regions of about 50% of human genes, resulting in formation of 5-methylcytosine (5mC) [62, 64]. Cytosine methylation in these promoter regions leads to the recruitment of corepressor complexes, resulting in reduced gene expression [4]. Both hypo- and hypermethylation of CpG islands at different locations have been associated with the development of leukemia [62]. Hypermethylation of transcriptional enhancers and promoters of protein coding and non-coding genes is observed in different cancer types including AML [65]. These genes are associated with cell cycle control, DNA repair, apoptosis, and signaling pathways [66–68]. For instance, the results of a cohort study including 344 AML patients indicated that downregulation of genome-wide gene expression was found to be linked to hypermethylation of the genes in the majority of patients and distinct methylation profiles could be observed in different AML subtypes. However, DNA methylation is not likely to be the only factor for leukemogenesis [69].

#### DNA methyltransferases in hematopoiesis

DNA methyltransferases (DNMTs) are responsible for DNA methylation, which are encoded by *DNMT1*, *DNMT2*, *DNMT3A*, *DNMT3B*, and *DNMT3L* genes in the human genome [70]. DNMT1, DNMT3A, and DNMT3B possess functional methylation activities. DNMT3A and DNMT3B called de novo methyltransferases are responsible for methylation of several CpG islands while DNMT1, the maintenance methyltransferase, shows a higher affinity for hemi-methylated CpG dinucleotides [70–72]. DNMT1 level reaches the highest at the early S phase, methylating the newly synthesized CpG sites located opposite to methylated CpGs on the mother strand during the S phase of DNA replication, ensuring highly accurate maintenance of methylation patterns and decreases to its lowest level at G1 phase [72, 73]. The dynamics of DNA methylation determines the fate of HSCs [74, 75]. Although CpG methylation in HSCs and progenitor cells is low during normal differentiation process, CpG methylation is increased in the specific regions of the genes to be silenced that are not associated with the committed lineage to ensure proper differentiation [76]. For instance, loss of methylation of transcription factors like *PRDM16*, *MEIS1*, and *HOXA9* is important for myeloid differentiation [77]. The maintenance of DNMT1 is essential for HSC survival and development through controlling self-renewal and differentiation, and suppressing apoptosis [74, 78, 79]. HSCs express higher levels of DNMT1 and its loss disrupts the differentiation and self-renewal potential of HSCs and progenitor cells [79]. DNMT1 depletion in HSCs leads to decreased promoter methylation of myeloerythroid genes including *CD48*, *GATA1*, *C/EBPalpha*, and *ID2* resulting in reactivation of these genes, which hence induce skewed differentiation to myeloerythroid lineages with impaired lymphopoiesis [74, 80]. Similar to DNMT1, DNMT3A is also important for HSC self-renewal and differentiation [81]. DNMT3A-deficient HSCs in mice led to extensive repopulation of HSCs and declined the differentiation potential. Aberrant methylation pattern was observed in differentiation-related genes such as *FLK2*, *PU.1*, and *IKAROS*. Also, multipotency-related genes including *RUNX1*, *GATA3*, *PBX1*, and *CDKN1A* were hypomethylated [81–83]. Loss of DNMT3B appears to have similar phenotype to DNMT3A loss but the effects are milder [84].

#### DNA methyltransferases in FLT3-ITD AML

DNMTs are involved in cancer initiation by its increased or decreased expression resulting in hyper- or hypomethylation, respectively. Therefore, they regulate several tumor suppressor genes and oncogenes including *BRCA1* and *TGFβ* in different cancer types and loss of DNMTs cause demethylation and re-expression of several tumor suppressor genes [85–87]. DNMT1 also maintains genomic stability through the interaction with DNA damage repair (DDR) systems that its disruption may contribute to cancer development. There are several studies showing hypermethylation of DDR genes in different cancer types [88–90]. In 2002, Di Croce provided the first mechanistic proof connecting genetic and epigenetic alterations in leukemogenesis suggesting that abnormal methylation of the genome plays a role in the early stages of tumorigenesis [91]. Di Croce found that PML-RAR fusion protein, an oncogenic transcription factor, recruited DNMT1 and DNMT3A to retinoic acid receptor beta 2 leading to its methylation and subsequent gene silencing in acute promyelocytic leukemia (APL) [91]. The interaction of DNMT1 with STAT3 and HDAC1 resulted in hypermethylation of SHP-1 tyrosine phosphatase in cutaneous T cell lymphoma [92]. The interaction of DNMT3A and DNMT3B with PU.1 in murine hematopoietic progenitor cells resulted in methylation of *p16* tumor suppressor gene [93]. These studies suggest that DNMTs can be recruited by oncogenic transcription factors to CpG sites of tumor suppressor genes leading to aberrant methylation and silencing of the genes involved in tumorigenesis. Moreover, these three DNMTs were reported to be overexpressed in AML cells [94].

*DNMT3B* mutations are rare in AML and de novo methylation activity is mainly due to DNMT3A in AML cells [95, 96]. *DNMT3A* mutations can be missense, nonsense, frame-shift, and splice-site mutation. The most common mutation in *DNMT3A* is at a single amino acid residue which is arginine 882 [97]. HSCs with *DNMT3A* mutation undergo a pre-leukemic state with increased self-renewal which results in dysregulated DNA methylation [83, 98, 99]. This aberrant methylation pattern could be important to detect AML with no clinical indication [95]. Malignant transformation of HSCs in pre-leukemic state occurs by acquiring additional genetic alterations including *FLT3*, *NPM1*, and *IDH1* mutations [97]. Moreover, this aberrant methylation in AML results in decreased expression of miRNA-370, a tumor suppressor, and increased expression of FOXM1, a tumor-promoting factor. Furthermore, dysregulated miRNAs in AML post-transcriptionally regulates *DNMT1* which might lead to overexpression of DNMT1 [100]. Development and relapse of AML can be triggered by DNMT1 overexpression through hypermethylation of tumor suppressor genes. For instance, spalt-like transcription factor 4 (SALL4), which is a key molecule in hematopoiesis and leukemogenesis, leads to recruitment of DNMT1 to its promoter to block self-repression of HSCs [101–103]. Fatty acid-binding protein 4 (FABP4) also regulates DNMT1 expression by inducing IL-6/STAT3 axis promoting AML aggressiveness [104].

There are limited mechanistic studies on the role of DNMTs particularly in *FLT3-ITD*-positive AML. *SHP-1* is a tumor suppressor gene negatively regulating JAK/STAT signaling which has an essential role in regulation of immune response, cell growth, and differentiation, therefore also in pathogenesis of AML. Mechanistically in *FLT3-ITD*-positive AML, upregulation of DNMT1, DNMT3A, and DNMT3B hypermethylate *SHP-1* result in silenced *SHP-1* and aberrant activation of STAT3 [105, 106]. Other tumor suppressor genes negatively controlling JAK/STAT pathway including *SOCS-1* and *SOCS-3* were also found to be silenced via promoter hypermethylation by DNMT1 [105]. Inhibition of DNMTs led to increased re-expression of tumor suppressor genes including *CDKN2B*, *BIM*, and *CDKN2A* in *FLT3-ITD*-positive AML [107]. The gene expression signature of *FLT3-ITD/DNMT3A* co-mutated murine models showed that *HOXB3*, *CDKN2C*, *MYC1*, *ZFP521*, and *MEIS1* genes, some of which are related to HSC self-renewal and differentiation, were upregulated compared to wild-type murine models [108]. Another study showed that *FLT3-ITD* mutation together with *DNMT3A* mutation or deletion in mice caused resistance to PARP inhibitor [109]. High expression of DNMT3B was associated with low expression of MTSS1, a tumor suppressor, and decreased OS, while low expression of DNMT3B is linked to high expression of MTSS1 and increased OS. Moreover, in murine AML cells overexpressing *FLT3-ITD*, MTSS1 was found to be downregulated which might be linked to DNMT3B overexpression [110].

#### Ten-eleven-translocation enzymes in hematopoiesis

Another epigenetic regulator includes ten-eleven-translocation (TET) enzymes which catalyze the oxidation of 5mC to form 5-hydroxymethylcytosine (5hmC) [111]. These enzymes are crucial for both active and passive DNA demethylation influencing the binding and recruitment of chromatin regulators [112]. Active DNA methylation is carried out by oxidation of 5hmC to 5-formylcytosine and 5-carboxylcytosine which are directly recognized by thymine DNA glycosylase-mediated base-excision repair to produce unmethylated cytosine [113]. While TET1 and TET2 play a role in oxidation of 5mC, TET3 does not modify the DNA but it does regulate the expression level of TET2 [114], of which the enzymatic activity is important for myelopoiesis and noncatalytic activity is needed for HSC self-renewal and lymphopoiesis [115]. Moreover, 5hmC can be recognized by specific transcription factors (e.g., MECP2, the MBD3/NURD complex, UHRF1, UHRF2, SALL1/SALL4, PRMT1, RBM14, and WDR76) to induce gene expression [116, 117]. Also, TET proteins act as tumor repressor by maintaining genomic stability [113]. TET proteins mark the damaged DNA sites and control the expression of DNA repair genes including *RAD50*, *BRCA1*, *RAD51*, *BRCA2*, and *TP53BP1* [118]. SMAD nuclear interacting protein 1 (SNIP1) is another TET2 interactor during DNA damage response. SNIP1 regulates c-MYC target genes participating in apoptosis by recruiting TET2 to the promoter region of c-MYC target genes [119]. Loss of *TET1/TET2* or *TET2/TET3* results in impaired DNA repair, suggesting that it promotes genomic stability. TET2 cooperates with TET1 and TET3 [120–123]. Consequently, loss of *TET2* results in reduced 5hmC marks at the damaged regions impairing the DNA repair [118, 120, 124, 125].

As DNMTs, TET protein family is also important for normal hematopoiesis [95]. The regulation of epigenetic landscape in hematopoietic stem/progenitor cells (HSPCs) by TET family controls the phases of lineage commitment at various differentiation stages [112, 120, 126–128]. Therefore, TET proteins collaborate with lineage-specific transcription factors [129–131]. TET1 deficiency in HSCs of mice led to skewed differentiation into B cell lineage showing that TET1 is important for lymphoid differentiation [132]. *TET3* deletion in mice resulted in increased number of HSCs without disturbing the frequency of differentiated cells [132]. TET2 is vastly expressed in HSCs but its expression is reduced after differentiation [133]. During myelopoiesis, C/EBPalpha activates TET2 and in cooperation with PU.1 and RUNX1 transcription factors, it is recruited to myeloid target genes including *KLF4*, *CHD7*, *JUN*, and *SMAD3* [131, 134–137]. TET2 also regulates the genomic accessibility of erythroid transcription factors including GATA1, SCL, and KLF1 [129, 130]. Deletion of *TET2* in mice enhanced the self-renewal capacity of HSCs by increasing the expression of MEIS1 and EVI1, and resulted in methylated erythroid transcription factor binding sites to block erythroid differentiation [137, 138]. Therefore, *TET2* disruption shifted differentiation toward myeloid lineages [137]. Not only homozygous but also heterozygous disruption of *TET2* enhanced the self-renewal capacity and myeloid differentiation showing that haploinsufficiency of *TET2* is enough to disturb HSCs homeostasis [112, 128, 139, 140]. Moreover, miRNA-22 and CXXC-containing protein IDAX target TET2 for degradation. Overexpression of miRNA-22 and CXXC-containing protein IDAX also caused increased self-renewal and defective differentiation of HSCs [134, 141, 142].

#### Ten-eleven-translocation enzymes in FLT3-ITD AML

*TET2* mutations are considerably common in hematological malignancies among three TET genes and *TET3* alterations are the least common [143, 144]. In myeloid malignancies, TET1 has a tumor-promoting role. Especially in AML with MLL-fusion proteins, TET1 overexpression has been observed [145]. TET1 exerts an oncogenic role in AML as it upregulates the expression of oncogenes including *HOXA9*, *MEIS1*, and *PBX3* [145] and downregulates the tumor suppressor targets such as miR-22 [146].

miR-22 represses CRTC1, FLT3, and MYCBP in *FLT3-ITD* human and mouse leukemic cells. Upregulation of TET1 represses miR-22 which eventually leads to aberrant activation of CREB, FLT3, and MYC pathway resulting in increased expression of oncogenic downstream targets [147]. High TET1 expression also regulates important oncogenic pathways as targeting STAT/TET1 axis proposed as a targeted therapeutic strategy in TET1 overexpressed AML [145, 148]. TET3 regulates the genes involved in AML-associated genes, glucose metabolism pathways, and STAT5A signaling pathway. Therefore, overexpression of TET3 promotes AML progression through epigenetic regulation of glucose metabolism and leukemic stem cell–associated pathways [149]. *TET2* mutations can be deletions, nonsense, and missense mutations at highly conserved residues inactivating the enzyme [150, 151]. *TET2* mutations are associated with reduced 5hmC levels, increased DNA methylation, and therefore increased epigenetic silencing [152]. TET2 controls the self-renewal of HSCs; hence, its loss in mouse model resulted in expansion of stem cells with increased repopulation ability [138, 146]. Consequently, *TET2* mutations in HSCs leads to shift into pre-leukemic state which disrupts the ability of HSCs to differentiate into mature blood cells. *TET2* mutations are considered early event in myeloid malignancies and additional mutations are needed for the development of full-blown leukemia [83, 98, 153]. *TET2*-mutated myeloid malignancies tend to have more mutational events than the *TET2* wild-type malignancies suggesting that loss of TET2 causes hypermutagenicity [123]. Mutations in *TET2* gene have been found in different hematologic malignancies including CMML, lymphoma, AML, MDS, and MPN [133]. Although the exact role of *TET2* mutations and which genes and pathways it regulates is not fully understood in AML, in vitro studies and animal models suggest that loss of catalytic function of TET2 may contribute to leukemogenesis due to disruption of the cell renewal control, but its sole mutations are insufficient to induce AML [112, 152].

AML patients with *TET2* mutations showed aberrant methylation and expression profiles of *SRSF2*, *ASXL1*, *RUNX1*, *DNMT3A*, *FLT3-ITD*, *C/EBPalpha*, and *JAK2* compared to *TET2* wild-type AML patients [154]. Although *TET2* mutation alone is not enough to induce leukemia, TET2 loss in combination with *FLT3-ITD* mutation is found to be sufficient to induce AML in vivo [152, 155]. Moreover, survival of these *TET2 ( −)/FLT3-ITD (* +*)* mice was significantly reduced compared to *FLT3-ITD* single mutated mice. Loss of TET2 in *FLT3-ITD*-mutated mice model resulted in reduced expression of *GATA2*, which is a regulatory gene in hematopoiesis and differentiation. Re-expression of GATA2 leads to restoration of differentiation and attenuates leukemogenesis [152]. *TET2/FLT3-ITD* co-mutation is also associated with the increased expression of long non-coding RNA (lnc) MORRBIDD specific to myeloid cells to regulate the lifespan, and loss of MORBID in *TET2/FLT3-ITD*-mutated mice model caused increased expression of BIM resulting in apoptosis and attenuated disease progression [156]. AML cells harboring both *TET2* and *FLT3-ITD* mutations showed hypermethylation profiles in the regulatory elements of the genes including *ID1*, *GATA1*, *MPL*, and *SOCS2* involved in self-renewal and differentiation [152].

#### Isocitrate dehydrogenases in hematopoiesis

Isocitrate dehydrogenases (IDHs) are NADP^+^-dependent enzymes catalyzing the conversion of isocitrate to a-ketoglutarate (a-KG) via oxidative decarboxylation within the krebs cycle [111, 157, 158]. The conversion of isocitrate to a-KG by IDH1/2 generates a crucial reducing agent, NADPH, which plays a pivotal role in regulating cellular defense mechanisms against oxidative stress through reduction of glutamine metabolism [159]. During this process, cells produce citrate and acetyl-CoA to sustain lipid metabolism and promote cellular growth under hypoxic conditions [160]. a-KG also binds to JmjC domain-containing histone demethylases (JmjC KDMs), TET2 and EGLN family of prolyl hydroxylases (PHDs), and ALKB homolog (ALKBH) DNA repair enzymes which have crucial roles in histone methylation, DNA methylation, and DNA repair, respectively [161, 162]. Hence, loss of *IDH1/2* results in impaired detoxification mechanism and aberrant methylation leading to increased DNA damage and genome instability in cancer cells [163].

IDH1 is primarily located in the cytoplasm and peroxisomes, while IDH2 is localized in mitochondria [164]. IDH3 is the third isozyme located in the mitochondria; however, it has not been defined as mutated in cancer [165]. *IDH1/2* mutations are heterozygous missense mutations in a single R residue within the active site of the enzyme [166]. Three conserved arginine residues, *R132* for *IDH1* and *R172* and *R140* for *IDH2*, are commonly mutated with wide-range of substitutions including polar (H, C, K, S, T, Q) and bulky nonpolar (W, V, M) amino acids [158]. The most common substitutions include *IDH1 R132H*, *IDH1 R132C*, *IDH2 R172K*, *IDH2 172 M*, and *R140Q* [167]. These mutations change the structure of the enzyme leading to reduced affinity to isocitrate and increased affinity to a-KG and NADPH to produce 2-hydroxyglutarate (2-HG) and NADP^+^ [168]. 2-HG disrupts metabolic processes and suppresses the krebs cycle, reducing the availability of α-ketoglutarate [169]. In cancer cells, *IDH1/2* mutations lead to excessive accumulation of 2-HG, which is an oncometabolite inducing biochemical and epigenetic alterations though competitive inhibition of a-KG-dependent enzymes including KDMs, PHDs, TETs, and ALKBH DNA repairs enzymes [167]. For instance, inhibition of TET2 by 2-HG leads to increased 5mC levels; hence, *IDH1/2* and *TET2* mutations are mutually exclusive showing significant overlap methylation signatures [111]. *IDH1 R132H* mutant allele expression in mice mirrored many aspects of *TET2* mutation and led to an increase in total 5-mC amount in HSPC compartment and bone marrow [114, 170]. Moreover, hypermethylation signatures in *IDH1/2* mutant AML patient samples and *TET2* mutant AML patient samples overlap and 93% of the genes which were overexpressed in *TET2*-mutated samples were also overexpressed in *IDH1/2* mutated samples [111].

Expression of *IDH2 R140Q* mutation in murine bone marrow cells inhibited myeloid differentiation and caused accumulation of immature progenitor cells [111]. Furthermore, expression of either *IDH2 R140Q* or *IDH1 R132H* in TF-1 human erythroleukemia cell line blocked the differentiation [171–173]. Retroviral transfection of CKIT^+^ with *IDH2 140Q* and *IDH2 R172K* resulted in decreased number of myeloid progenitor cells and HSC accumulation [174]. Moreover, *IDH1 R132H* or *IDH2 R140Q* overexpression in HSCs led to extramedullary hematopoiesis and splenomegaly showing the presence of a myeloproliferative disease [175–177]. Mechanistically, TET2 is inhibited by 2-HG; therefore, TET2 cannot regulate PU.1 and WT1 target genes, leading to transcriptional repression [178, 179]. Not only PU.1 target genes but also *GATA1* and *GATA2* binding sites were 5mC enriched in *IDH1/IDH2* mutant cells [111, 178, 180]. *IDH1*-mutated cells displayed overexpression of *HOXA* and *HOXB* cluster genes altering the differentiation which was associated with altered expression of RUNX1, PU.1, GATA1, and C/EBPalpha transcription factors [181]. In addition to TET2 inhibition, KDM inhibition by 2-HG results in decreased histone demethylation leading to differentiation blockage and promoted self-renewal of HSCs [162]. Hence, *IDH1/2* mutations and 2-HG accumulation result in inhibition of blocked differentiation of HSCs and enhanced self-renewal through inhibition of DNA and histone demethylation [182]. 2-HG inhibits ALKB family proteins in a similar manner to TET enzymes; therefore, inhibition of ALKB family proteins leads to reduced DNA repair and accumulated DNA damage which may lead to cancer development through mutations [183].

#### Isocitrate dehydrogenases in cancer

Inhibition of PHD, which are the regulatory proteins to degrade hypoxia-inducible factor 1a (HIF-1a), also promotes cancer development and progression [162]. In addition to aforementioned effects of accumulation of 2-HG, apoptosis and cell cycle regulation are also affected by the *IDH1/2* mutations. Mutant *IDH1* leads to suppression of *CDKN2A* and *CDKN2B* stimulating MAPK pathway and results in enhanced cell proliferation [176, 184]. Furthermore, 2-HG inhibits cytochrome-c oxygenase, a component of the mitochondrial electron transport chain involved in removal of reactive oxygen species. Although the precise mechanism remains unclear, this inhibition leads to elevated BCL-2 expression, thereby inhibiting apoptosis in leukemic cells [185]. 2-HG stabilizes NF-kB activation in bone marrow stromal cells through ERK kinase pathway; hence, active NF-kB activates IL-6 and IL-8 secretion inducing AML cell proliferation [183]. 2-HG also induces hypermethylation signatures on the WNT inhibitory signals leading to increased stemness [186].

Despite the epigenetic and cellular changes caused by *IDH1/2* mutations, expression of mutant *IDH1/2* enzymes does not induce leukemic transformation [182]. Mouse models expressing mutant IDH1 or IDH2 showed similar patterns to those found in AML patients such as increased early hematopoietic progenitors, splenomegaly, anemia, hypermethylated DNA, and histone signatures. However, the mouse models did not develop full-blown leukemia [175]. Therefore, *IDH1/2* mutations are considered early events in leukemogenesis and additional mutations including *FLT3*, *MEIS1A*, *HOXA9*, and *NRAS* are crucial to drive leukemogenesis [174, 177].

### Prognostic impact of DNMTs in FLT3-ITD AML

Mutations in *DNMT3A* is identified in 15–25% of AML patients [62] and 36–44% of *FLT3-ITD-*positive AML patients carry concurrent *DNMT3A* mutations [187]. Although the role of mutant *DNMT3A* in leukemia development remains unclear, one hypothesis proposes that mutant *DNMT3A* has a dominant negative effect over wild-type *DNMT3A* [98]. Notably, *R882H* mutation in AML cells has been reported to have a significant reduction in de novo methyltransferase activity and increased proliferation [96, 188]. Co-occurrence of *DNMT3A* mutations with *FLT3-ITD* mutation is found to be indicative of a poorer prognosis [97, 189]. The peripheral white blood cell count is significantly higher in *DNMT3A/FLT3-ITD* co-mutated AML patients compared to *FLT3-ITD* AML patients and *DNMT3A/FLT3-ITD* mutations results in higher burden of disease compared to only *DNMT3A*-mutated AML patients [190, 191]. Concurrent *DNMT3A* and *FLT3-ITD* mutation in AML patients is related to poorer OS and worse outcomes after chemotherapy compared to single mutated *FLT3-ITD* or *DNMT3A* AML patients [190–194] The effectiveness of chemotherapy is also decreased in *DNMT3A/FLT3-ITD*-mutated patient group [191]. Moreover, patients carrying persistent *DNMT3A/FLT3-ITD* mutations had the highest rate of relapse after induction therapy [195]. Furthermore, early presence of *DNMT3A* mutations is found to be linked to a higher incidence of *FLT3-ITD*-positive clones at relapse [64, 196]. While *DNMT3A* mutations are the most studied ones in FLT3-ITD AML, there are limited studies related to the roles of other DNMTs. High DNMT1 expression was correlated with p15 methylation in *FLT3-ITD* AML and DNMT1 inhibition caused upregulation of *p15* and *p16* tumor suppressor genes [107]. Another report showed that high DNMT3B expression was observed in *FLT3-ITD* AML patients compared to *FLT3-WT* AML patients, which was associated with poor prognosis [110].

### Prognostic impact of TETs in FLT3-ITD AML

*TET1* mutations are found in 1–5% AML patients [17]. As the most mutated TET family protein is TET2, mutations in *TET2* gene are observed in 7 to 23% of AML patients particularly with a normal karyotype and are associated with poorer OS and reduced response to chemotherapy [62, 64, 197]. One study demonstrated that AML patients with homozygous *TET2* mutations showed inferior event-free survival (EFS) and higher relapse rate compared to heterozygous *TET2*-mutated AML patients [198]. Moreover, the presence of *TET2* mutations together with *FLT3* mutations indicates an adverse outcome [199–201]. Patients with *TET2/FLT3-ITD* double mutation had lower the 3-year OS (37.9% vs 25%), DFS (48.9% vs 16.7%), and EFS (27.8% vs 16.7%) compared to patients with *FLT3-ITD* mutation [202]. Co-occurrence of *TET2* mutations with *FLT3-ITD* mutations may result in the development of leukemia inducing synergistic gain-of-function effects on DNA methylation, therefore gene expression. Moreover, blocking only FLT3 signaling in *TET2/FLT3-ITD*-mutated AML is not enough to restore mutated TET2 activity. Therefore, methylated CpGs cannot be reversed, presenting a potential resistance mechanism to FLT3 inhibitor monotherapy [152]. However, deletion of *TET2* from *FLT3-ITD*-mutated cells increased sensitivity toward PARP inhibitor or PARP inhibitor plus quizartinib therapy, while deletion of *DNMT3A* caused resistance compared to *FLT3-ITD*-mutated cells [109]. In contrary, in *TET2/FLT3-ITD*-mutated mice model, CXCR4 and CXCL12 expressions were increased compared to *FLT3-ITD* mice causing resistance to chemotherapy and FLT3 inhibitor [155]. In a de novo study, high frequency of *FLT3-ITD* mutations at relapse was associated with *TET2* or *IDH1/2* mutations suggesting that mutations in epigenetic regulators in AML may induce *FLT3-ITD* mutations through genetic instability and it may lead to relapse and resistance to therapy [196].

### Prognostic impact of IDHs in FLT3-ITD AML

The frequencies of *IDH1* and *IDH2* mutations in AML are 7–14% and 8–19%, respectively [165]. *IDH1/2* mutations are exclusively heterozygous and are generally found in AML patients with normal cytogenetics [17, 200, 203]. The prevalence of *IDH1/2* mutations is higher in older AML patients carrying intermediate risk [17]. Moreover, patients carrying *IDH1/2* mutations frequently have higher platelet count, higher peripheral blast, and bone marrow percentages compared to wild-type *IDH1/2* AML patients [204–206]. IDH mutations are mostly considered an early event in carcinogenesis and may remain after chemotherapy or at relapse [207–209]. Although AML patients with *IDH1* mutations and AML patients with *IDH2* mutations show a significant overlap in the differentially expressed genes, in a meta-analysis including 12,747 patients, it was reported that *IDH1* mutation was associated with inferior OS and EFS while *IDH2* mutation was associated with favorable OS [210]. Another meta-analysis showed that the most frequent concurrent mutation is *NPM1* followed by *FLT3-ITD* [206]. Moreover, *IDH1* and *IDH2* mutations co-occur in 15–27% and 8–30% of AML patient with *FLT3-ITD*, respectively [211]. Furthermore, co-occurrence of *FLT3-ITD* and *IDH1* mutation is considered a negative prognostic factor and is associated with shorter OS and EFS [210].

### Targeting DNA methylome in FLT3-ITD AML

Targeting DNA methylation in AML has gained significant attention, given the fact that it represents a potential therapeutic strategy to reverse the epigenetic dysregulations that contribute to disease progression, drug resistance, and inferior prognostic impact in the presence of *FLT3-ITD* mutations.

Azacitidine and decitabine are pyrimidine analogs approved for the treatment of AML, resulting in low blast count. These compounds function as the inhibitors of DNMTs to reverse DNA hypermethylation, therefore restoring the expression of critical genes including tumor suppressor genes [212]. Azacitidine incorporates into RNA and to a lesser extent into DNA while decitabine only incorporates into DNA. DNA incorporation leads to formation of covalent bonds with DNA methyltransferases, hence blocking DNA hypermethylation [213, 214]. Although there are several studies on decitabine or azacitidine for AML treatment, studies of these drugs as a single agent on *FLT3-ITD*-mutated AML are limited. Hu and colleagues showed that decitabine, as a single agent, on MV4-11 and MOLM-13 *FLT3-ITD* AML cells, induced C/EBP alpha-PU.1 pathway, which is required for myeloid differentiation, leading to downregulation of FLT3 and induction of apoptosis. In another study, *FLT3-ITD* + */TET2* mutant mice model treated with azacitidine, which resulted in normalization in peripheral blood cell counts and splenomegaly, decreased total white blood cell count and spleen weight. Also, treatment with azacitidine reduced the aberrant DNA methylation in the stem-progenitor cells. Additionally, azacitidine treatment restored the myeloid maturation toward normal neutrophil populations [215]. In mouse xenograft models of *FLT3-ITD* AML, decitabine was effective to reduce the tumor volume [216]. Treatment of CEP-701, a TKI, resistant-MV4-11 cell line with azacitidine, resulted in re-expression of *SHP-1*, a tumor suppressor gene and negative regulator of STAT3, leading to suppression of STAT3 and induction of apoptosis [106]. Another study evaluating the effects of decitabine and azacitidine showed that both azacitidine and decitabine treatment resulted in DNA double-strand breaks and subsequent activation of p53 in MOLM-13 cells. Also, both agents reduced the expression of *TERT*, *BCL-2*, and *MYC* oncogenes in these cells [217]. In a post hoc analysis of QUAZAR AML-001 phase 3 trial (NCT01757535), 66 patients with *FLT3*-mutated AML (46 patients with *FLT3-ITD*) were treated with oral azacitidine and the median OS was prolonged in *FLT3*-mutated AML patients compared to the placebo group (28.2 months vs 9.7 months). Relapse-free survival (RFS) was also improved in *FLT3*-mutated AML patients with 23.1 months compared to placebo (4.6 months) [218].

Targeting DNMT in *FLT3-ITD* AML is also achieved through modulation of DNMT regulators. Sp1 is a zinc finger transcription factor which binds to the promoter region of *DNMT1* and p65 is NF-κB signaling component which interplays with Sp1 for DNMT1 transactivation in *FLT3-ITD* AML. Therefore, disruption of Sp1/NF-κB complex impairs the expression of DNMT1 leading to DNA hypomethylation [219–221]. Bortezomib, a proteasome inhibitor, is used to reduce Sp1 levels in MV4-11 cells resulting in decreased DNMT1 expression, therefore leading to lower global genomic DNA methylation compared to the control [221]. Thymoquinone is a natural product with anti-cancer activity which decreases the Sp1 levels. It decreases DNMT1 and DNMT3A expression levels leading to downregulation of KIT, FLT3, STAT5, and AKT in MV4-11 cells [220]. Moreover, treatment of MV4-11 cells with either thymoquinone or azacitidine leads to decreased methylation levels on the promoter regions of *SOCS-1*, *SHP-1*, and *SOCS-3* tumor suppressor genes, causing increased expression of these genes and decreased expression of FLT3-ITD, JAK2, STAT3, and STAT5 [105].

Two agents are recently approved to target IDH mutations [222]. Enasidenib is a selective allosteric inhibitor of IDH2 which binds to open conformation of the enzyme and stabilizes it, thereby blocking the conversion of a-KG to 2-HG [223]. Ivosidenib is a reversible allosteric competitive inhibitor of IDH1, competing with the essential cofactor, magnesium ion, to bind to IDH1, which prevents the formation of a catalytically active site [224]. Enasidenib therapy in *FLT3-ITD* + */IDH2 R140Q* double mutated mice model decreased the 2-HG serum levels and induced demethylation of the hypermethylated promoters in leukemia-derived cells. Moreover, treatment with enasidenib normalized the myeloid maturation [215]. In clinical trials, enasidenib alone was not effective for the patients with *FLT3* and *IDH* co-mutations [225].

### Combinational therapies including clinically approved FLT3Is and epi-drugs

**Fig. 2:**
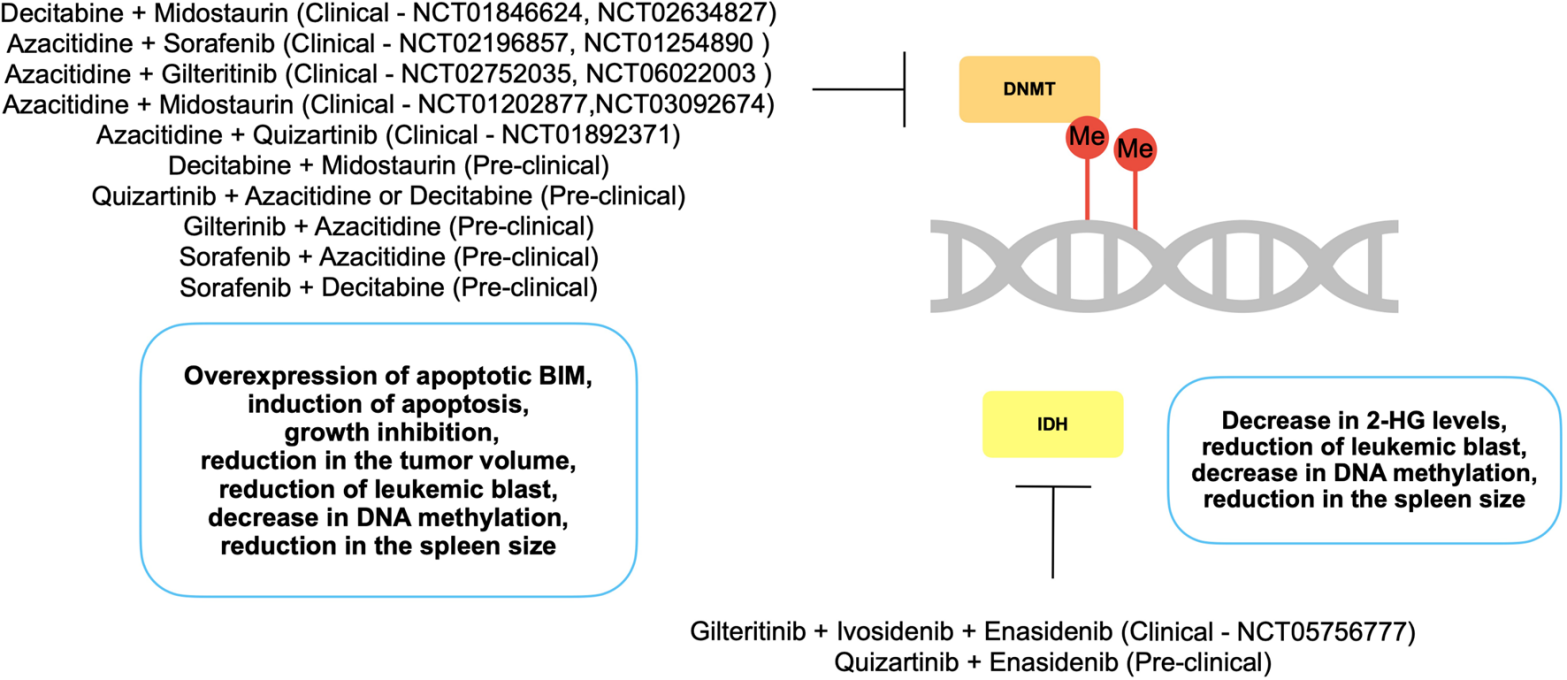
Combinational approaches involving epi-drugs and FLT3Is in pre-clinical and clinical studies in FLT3-ITD AML.

The rationale behind combining FLT3Is with epigenetic drugs lies in the potential synergy they could create to enhance FLT3I’s activity especially when FLT3I resistance occurs. FLT3 inhibitors can directly target the cancer cells with *FLT3* mutations while epigenetic drugs can modify the epigenetic landscape of the cells, potentially enhancing the effectiveness of FLT3 inhibition. Several pre-clinical and clinical studies have explored the combination of FLT3Is and epi-drugs in *FLT3-ITD* AML treatment (Tables 1 and 2, Fig. 2).

**Table 1:** Preclinical studies targeting DNA methylation regulators either alone or in combination with clinically approved FLT3Is in FLT3-ITD AML

Inhibitors	Experimental model	Study outcomes	References
Decitabine (DNMT inhibitor)	In vitro In vivo	Induction of C/EBPa-PU.1 pathway, Downregulation of FLT3, induction of apoptosis, reduction of tumor volume	[216]
Azacitidine (DNMT inhibitor)	In vitro	Re-expression of SHP-1, suppression of STAT3, induction of apoptosis	[106]
Azacitidine or decitabine	In vitro	Activation of p53, reduced expression of TERT, BCL-2 and MYC	[217]
Enasidenib (IDH2 inhibitor) + quizartinib	In vivo	Reduction of the blast population and reduced spleen size	[226]
Decitabine + midostaurin	In vitro	Overexpression of BIM, induction of apoptosis	[227]
Quizartinib + azacitidine or decitabine	In vitro	Synergistic growth inhibition	[228]
Gilteritinib + azacitidine	In vitro In vivo	Induction of apoptosis, decreased tumor volume	[229]
Sorafenib + azacitidine	In vitro In vivo	Inhibition of cell growth, induction of apoptosis, decreased tumor volume	[230]
Sorafenib + decitabine	In vitro	Synergistic growth inhibition	[231]
Quizartinib + enasidenib	In vivo	Suppression of malignant clones, reduction of leukocytosis	[215]

**Table 2:** Key clinical trials of novel epigenetic therapies in combination with approved FLT3Is in FLT3-ITD AML

Phase/clinical trial number	Intervention	Patient group	Response	Status
Phase II NCT01846624	Decitabine + midostaurin	Elderly patients with newly diagnosed FLT3-ITD/TKD-positive AML	CR after induction therapy: 66.7% 1-year OS: 83.3% PFS: 0%	Terminated
Phase II NCT02634827	Decitabine + midostaurin	Older patients with newly diagnosed AML with FLT3 mutation	No results posted	Terminated
Phase I/II NCT01202877	Azacitidine + midostaurin	Refractory or relapsed AML patients with FLT3 mutations	OR rate in phase I: 14% OR rate in phase II: 30%	Completed
Phase II/III NCT03092674	Azacitidine + midostaurin	Newly diagnosed older AML patients with FLT3 mutations	No results posted	Active, not recruiting
Phase I/II NCT01892371	Azacitidine + quizartinib	Relapsed or refractory FLT3-ITD AML or Myelodysplastic Syndrome (MDS)	CRc in R/R AML: 64% OS in R/R AML: 12.8 months CRc in frontline therapy: 87% OS in frontline therapy: 19.2 months	Completed
Phase I/II NCT01254890	Azacitidine + sorafenib	Relapsed or refractory AML and MDS patients with FLT3 mutations	CRc rate: 46% CR rate: 16%	Completed
Phase II NCT06022003	Azacitidine + gilteritinib	Refractory or relapsed AML patients with FLT3 mutations	No results posted	Recruiting
Phase II NCT02196857	Azacitidine + sorafenib	AML and high risk MDS patients with FLT3-ITD mutation	CR rate: 25%	Completed
Phase I NCT05756777	Gilteritinib + ivosidenib + enasidenib	Relapsed/refractory AML patients with FLT3/IDH1 or FLT3/IDH2 mutations	No results posted	Recruiting
Phase III NCT02752035	Gilteritinib + azacitidine	Newly diagnosed AML with FLT3 mutation in patients not eligible for intensive induction chemotherapy	CRc rate: 58.1% EFS: 4.5 months OS: 9.8 months	Active, not recruiting

*CR* complete remission, *CRc* composite complete remission, *EFS* event-free survival, *OS* overall survival, *PFS* progression-free survival, *OR* overall response

Sequential treatment with decitabine followed by midostaurin synergistically induced apoptosis through overexpression of apoptotic BIM in *FLT3-ITD* AML-positive MV4-11 and MOLM-13 cells [227]. Treatment of *FLT3-ITD* + */TET2*-mutant and *FLT3-ITD* + */IDH2 R140Q* double mutated mice models with quizartinib in combination with azacitidine or enasidenib, respectively, showed significant response compared to control or monotherapy, reducing leukemic blast in the bone marrow, spleen, and liver. Combination therapy decreased DNA methylation even more than the monotherapy [215]. *FLT3-ITD* and *IDH2 R172K* or *IDH2 R140Q* double mutated zebrafish model, when treated with quizartinib and enasidenib, showed reduction in the blast population and spleen size [226]. Treatment of MOLM-14 cells and newly diagnosed and relapsed FLT3-ITD-mutated AML patient–derived cells with quizartinib and azacitidine/decitabine resulted in synergistic growth inhibition. When sequential or simultaneous treatment with quizartinib and DNMT inhibition were compared, simultaneous DNMT inhibition and quizartinib treatment induced synergistic or additive effect compared to sequential treatment [228]. In another study, treatment of MV4-11 and MOLM-13 cells with gilteritinib and azacitidine resulted in induction of apoptosis, and in xenografted mouse tumor volume was significantly decreased in gilteritinib plus azacitidine–treated group compared to gilteritinib-treated group [229]. Similar to quizartinib and gilteritinib, the combination of azacitidine with sorafenib also synergistically inhibited MOLM-13 and MV4-11 cell growth, induced apoptosis, and decreased tumor volume in xenograft mouse model of *FLT3-ITD* AML. In another study, sorafenib is combined with decitabine to treat MV4-11 cells, resulting in growth inhibition and synergistic effect [231].

Quizartinib plus azacitidine treatment was given as a frontline or salvage therapy in a cohort of 38 *FLT3-ITD* AML patients. Eighty-seven percent of composite response (CRc) rate was achieved in the frontline-treated group and 67% of CRc was achieved in R/R AML patients [232]. When *FLT3-ITD* AML patients treated with azacitidine combined with sorafenib, allelic burden and *FLT3-ITD* clone size were significantly reduced and progression-free survival (PFS) was significantly increased compared to sorafenib treatment alone [230]. Patients who had relapsed after allo-SCT and had *FLT3-ITD* mutations treated with 5-azacitidine and sorafenib. While three out of 5 patients achieved CR, the remaining 2 patients experienced disease progression [233]. In a cohort of 40 *FLT3-ITD*-mutated AML patients, treatment with sorafenib plus azacitidine resulted in 46% of overall CRc rate. While previously untreated patients had 67% of response rate, it was lower for primary refractory or relapsed patients with 58% and 32%, respectively (NCT01254890) [234]. Another clinical trial, in which 27 newly diagnosed elderly (61–86 years) AML patients with *FLT3-ITD* mutation were treated with sorafenib plus azacitidine, resulted in 78% of overall response rate (ORR) suggesting that azacitidine and sorafenib combination is well tolerated and effective to treat older patients with *FLT3-ITD*-mutated AML who have not received previous treatment (NCT02196857) [235]. In a phase 3 trial, 123 patients with *FLT3-ITD-*positive AML who are not eligible for intensive chemotherapy were treated with gilteritinib plus azacitidine or azacitidine alone and CRc rates were 58.1% and 26.5% respectively showing no new significant safety issues compared to individual therapy (NCT02752035) [236••].

Murine model of *FLT3-ITD* and *IDH2* co-mutated AML showed that enasidenib treatment significantly reduced 2-HG levels and induced demethylation of hypermethylated CpG islands, but enasidenib alone was not enough to suppress the malignant clones. However, when combined with quizartinib, leukocytosis reduction was more effective compared to enasidenib monotherapy [215]. In a recent study, FLT3 and/or IDH inhibitors (FLT3Is and/or IDHIs) were administered as a single agent or in combination with cytotoxic chemotherapy (CCT) or low-intensity therapy (LIT) in 91 AML patients with FLT3-ITD/IDH1 or FLT3-ITD/IDH2 double mutations. The results demonstrated that a combination of a FLT3I with CCT or LIT was effective in patients with FLT3-ITD/IDH co-mutated disease in both the frontline and R/R settings [237].

## Conclusions

Understanding the roles of epigenetic regulators in normal hematopoiesis and in initiation and maintenance of hematopoietic malignancies including *FLT3-ITD* AML have paved the way for the development of epigenetically targeted therapies including small-molecule inhibitors of certain epigenetic regulators involved in DNA methylation (azacitidine and decitabine) and other epigenetic-related processes such as IDH-related modifications. Some of these therapeutic approaches have already undergone clinical trials alone or in combination with clinically approved FLT3Is including midostaurin, sorafenib, and gilteritinib which evaluate their efficacy, safety profile, and patients’ benefit as indicated in Table 2. However, majority of them is still infant passing through in vitro and in vivo experimental stages with promising outcomes (Table 1). On the other hand, it is quite important to define epigenetic alterations at the individual level for the development of specific epigenetic biomarkers to predict response to therapy and to identify epi-drugs with specific and durable outcomes in the near future for the treatment of *FLT3-ITD* AML patients. It would be assumed that combination of epigenetic therapy and FLT3Is could promise higher success rates as compared to epi-drugs or FLT3Is alone after revealing mechanisms of action of novel epigenetic alterations and carrying out larger randomized trials.

## Data Availability

No datasets were generated or analysed during the current study.
